# A Deep Learning Pipeline for Cell Segmentation and Viability Quantification in 3D Constructs From Fluorescence Microscopy Images

**DOI:** 10.1002/jemt.70170

**Published:** 2026-08-12

**Authors:** Federica Valtellina, Francesco Iannacci, Bianca Maria Colosimo, Mattia Sponchioni

**Affiliations:** ^1^ Department of Chemistry Materials and Chemical Engineering “Giulio Natta,” Politecnico di Milano Milano Italy; ^2^ Department of Mechanical Engineering Politecnico di Milano Milano Italy

**Keywords:** 3D bioprinting, automatic count, cell viability, in‐situ monitoring, machine learning

## Abstract

With the increasing adoption of 3D cell cultures and bioprinting in biomedical research, there is a growing demand for reliable and accurate monitoring methods. While fluorescence microscopy is widely accepted for 2D cell cultures, when introducing the third dimension it often suffers from signal attenuation and out‐of‐focus interference, making automated image analysis challenging. In this study, a hybrid pipeline that integrates deep learning and traditional image processing techniques is presented with the aim of establishing an automated tool for cell segmentation and viability assessment in 3D from fluorescence microscopy images. A *U* ^2^‐Net architecture was trained on 2D cell cultures for cell segmentation, while watershed‐based separation and intensity‐based classification were employed for viability detection. The model demonstrated accurate segmentation with minimal overfitting. Quantitative comparisons with manual counts and ImageJ‐based results confirmed its accuracy. We then demonstrated the possibility of extending this model to 3D constructs by tracking cell density and viability during time in the case of human umbilical vein endothelial cells bioprinted in gelatin methacrylate at different seeding densities, taken as case study. Automated counts closely matched manual ones, highlighting the method reliability and minimal invasiveness avoiding the need for sacrificing the construct. Hence, this approach provides a scalable, reproducible, and efficient alternative to manual counting in 3D environments, enabling high‐throughput analysis of complex fluorescence microscopy data.

## Highlights

- Hybrid pipeline based on the *U* ^2^‐Net architecture for the analysis of fluorescence microscopy images.
- Automated cell segmentation and viability assessment in 3D constructs.
- Nondestructive method allowing for in situ monitoring.
- Reliable results when benchmarked against manual counts and standard image analysis algorithms.
- Reliable tracking of cell density and viability during time in 3D‐bioprinted constructs.

## Graphical abstract

3D bioprinting requires advanced methods for cell localization and viability assessment. We developed a hybrid and automated pipeline based on the *U* ^2^‐Net architecture for the analysis of fluorescence microscopy images enabling cell segmentation and viability assessment. The model was used to track cell viability in 3D constructs demonstrating improved reliability compared to conventional software.

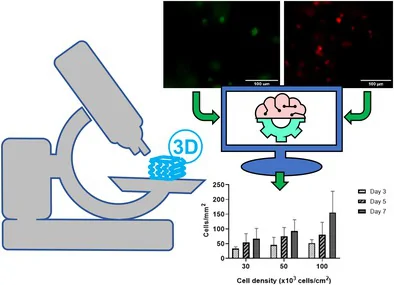

## 1 Introduction

In recent years, three‐dimensional (3D) cell cultures and bioprinted constructs have emerged as a promising technology in biomedical research due to their ability to better replicate the physiological and structural complexity of native tissues compared to traditional two‐dimensional (2D) monolayers (Sharma et al. 2023; Breslin and O’Driscoll 2013). In particular, bioprinting can be defined as the layer‐by‐layer deposition of biocompatible materials, mostly biopolymers, containing living cells and other biological factors on the basis of a computer‐aided design. This technology finds application in a variety of areas, ranging from tissue engineering to regenerative medicine, clinics, drug screening, and disease modeling (Ozbolat 2015; Ozbolat et al. 2016). Among a variety of 3D bioprinting strategies, extrusion‐based bioprinting is the most widely used due to its ability to deposit cells and biomaterials with high spatial precision and reduced mechanical and chemical stresses, facilitating the fabrication of complex, multicellular constructs (Salaris and Rosa 2019; Heinrich et al. 2019; Matai et al. 2019).

With the growing diffusion of 3D constructs, there is a corresponding need for reliable methods to assess cell behavior within these environments and track their viability and density over time. Fluorescence microscopy, particularly live/dead assays using dual fluorescent staining, remains the standard technique adopted for assessing cell viability. In fact, it offers good accuracy while being a nondestructive technique, hence suitable for in situ monitoring. However, the transition from 2D to 3D imaging presents new challenges. Image quality deteriorates in 3D samples due to reduced optical transparency, signal attenuation with depth, and increased out‐of‐focus interference, thus complicating quantitative analysis (Smith et al. 2010; Bikmulina et al. 2022). A recent work reported the in situ analysis of cell spatial distribution during the bioprinting of 3D constructs by fluorescence imaging, providing a promising solution to overcome the mentioned challenges, while at the same time automatizing the process (Margarita et al. 2025).

However, the accurate quantification of live and dead cells from fluorescence images remains a critical and often limiting step in experimental workflows (Piccinini et al. 2014; O'Brien et al. 2016; He et al. 2019). Manual counting is still commonly employed but it is time‐consuming, subjective, and not scalable for high‐throughput experiments. Semi‐automated methods exist, but they often rely on expensive instrumentation or require well‐separated cells and uniform signal intensity to be effective (Lavitt et al. 2021; De Boodt et al. 2013). Reliable cell quantification first requires accurate segmentation, differentiating cells from the background, which is highly sensitive to image features like brightness, contrast, and signal uniformity. This task becomes especially challenging in dense or noisy samples, where cells overlap or exhibit weak fluorescence signals. Although open‐source tools, such as ImageJ and CellProfiler, offer modular pipelines, frequently addressing specific segmentation problems, they require the combination of multiple techniques and a manual fine‐tuning of the corresponding parameters. Given the rigidity of the architecture, even small variations in image quality, such as changes in illumination, background noise, or staining intensity, can significantly degrade segmentation performance, often necessitating repeated and labor‐intensive adjustments (Wang et al. 2018). As a result, existing workflows tend to suffer from poor reproducibility, low transferability, and over‐ or underestimation under challenging conditions (De Boodt et al. 2013; Wang et al. 2018).

Recent advances in machine learning, particularly deep learning, have significantly improved the automation and accuracy of image analysis tasks. In biomedical imaging, convolutional neural networks (CNNs) have become widely adopted due to their ability to learn hierarchical and problem‐specific features directly from training data, eliminating the need for hand‐made features or prior assumptions (Lavitt et al. 2021; Moen et al. 2019; Alahmari et al. 2019). These models have shown strong performance across a wide range of computer vision tasks, such as classification, object detection, and segmentation, making them ideal candidates for analyzing complex fluorescence microscopy images (Moen et al. 2019; Alam and Islam 2019). Among CNN architectures, *U* ^2^‐Net has demonstrated strong performance in image segmentation tasks, being more and more considered for applications in biomedical image analysis (Wang et al. 2018; Yamashita et al. 2018; Li et al. 2024; Antonelli et al. 2022; Azad et al. 2024; Tan et al. 2026; Huo et al. 2025; Fang et al. 2024). This network has gained attention for its effectiveness in salient object detection (SOB) through a nested U‐structure that allows for high resolution while maintaining computational efficiency (Qin et al. 2020). However, deep learning models still face some difficulties when dealing with densely packed or connected cells, as accurately detecting thin boundaries is challenging with limited labeled data. The lack of standardized, diverse training datasets for different cell types and imaging conditions further limits the performance of these models. Recent studies have been proposed to overcome the issue of limited annotated datasets (Zhou et al. 2024; Zargari et al. 2024). A relevant example is the work from Çelebi et al., who demonstrated that representation‐level self‐supervised contrastive learning improved Mask R‐CNN‐based senescent cell segmentation in brightfield microscopy images, increasing downstream segmentation performance compared with the original segmentation model (Çelebi et al. 2024). However, common imaging artifacts, such as blurring, noise, and focus drift, can compromise segmentation quality, emphasizing the need for robust, adaptable solutions that perform reliably in experimental setups (Wang et al. 2018).

To address these challenges, this work proposes a fully automated pipeline for cell segmentation and viability assessment from fluorescence microscopy images in the specific case of human umbilical vein endothelial cells (HUVECs), chosen as a model system. The methodology consists of a two‐stage pipeline: (1) *U* ^2^‐Net‐based segmentation for accurate cell boundary detection, and (2) watershed‐based separation and intensity‐based classification for quantifying live and total cells. By integrating both deep learning and traditional algorithms, this hybrid approach mitigates the limitations of each of them: it achieves high segmentation accuracy while remaining adaptable to different imaging conditions without requiring extensive manual tuning or retraining. The pipeline developed in this work is schematized in Figure 1. We initially developed this tool from 2D HUVEC cultures, enabling facile handling and the acquisition of a robust dataset from brightfield and fluorescence microscopy images. In fact, the cells were transfected to express the green fluorescent protein (GFP) as free cytoplasmatic protein, which was taken as indicator of viable cells. In addition, before seeding, HUVECs were stained with Rhodamine B‐tagged polymer nanoparticles to provide a red fluorescence signal, taken to estimate the total cells (i.e., dead + live). These images were used for hyperparameter selection and model training. The comparison of the results from a validation dataset, excluded from the calibration stage, with manually counted cells confirmed the accuracy of the model in both segmentation and viability classification. We then demonstrated its translatability to 3D bioprinted constructs, where this model allowed the accurate tracking of cell density and viability for HUVECs in gelatin methacrylate structures in a fully automated way. Moreover, this protocol avoided the need for sacrificing the constructs, as it is required with traditional live/dead assays. Overall, the pipeline modular implementation allows for easy integration into a wide range of microscopy workflows. This of course may require dedicated model training based on the specific system used but the advantage would then be a significant reduction in user workload, enhancement of reproducibility, and high‐throughput fluorescence image analysis.

**FIGURE 1:**
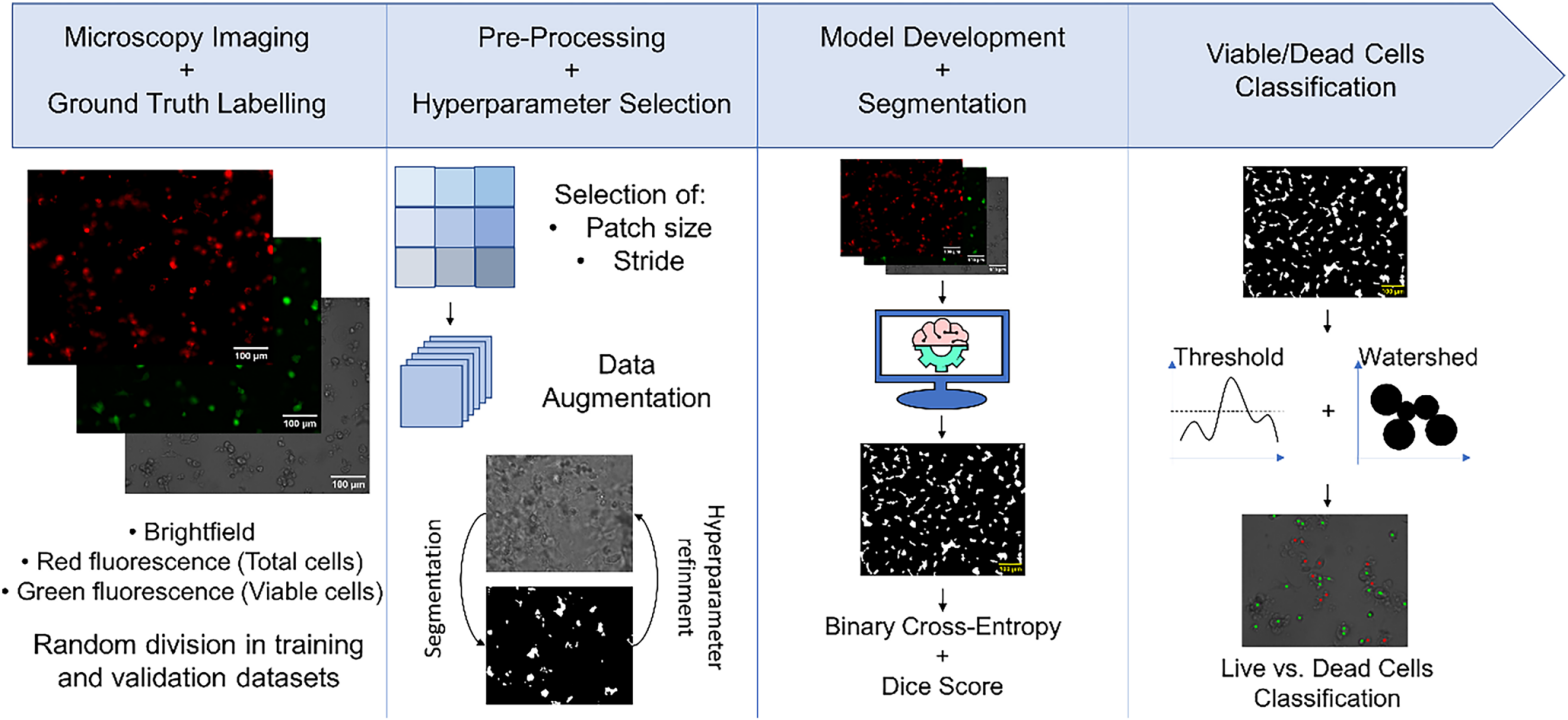
Schematic representation of the pipeline combining *U* ^2^‐Net cell segmentation and watershed separation and classification in live and dead cells from brightfield and fluorescence microscopy images.

## 2 Materials and Methods

### 2.1 Experimental Protocols

#### 2.1.1 Cell Culture and Maintenance

HUVECs transiently transfected with the turbo green fluorescent protein (turboGFP) vector were acquired from Innoprot (Spain), catalog number P20201, and used as a cell model. Cells were cultured in endothelial cell growth medium (PromoCell, Germany), supplemented with 1% w/v penicillin/streptomycin (Sigma‐Aldrich, St. Louis, USA) at 37°C, 5% v/v CO_2_, 95% humidity in a Forma Steri‐Cycle CO_2_ Incubator. Cells were used between passages 6 and 8. To enable dual fluorescence imaging, cells were treated with rhodamine B‐labeled polymethyl methacrylate nanoparticles at a concentration of 0.3 mg/mL, 24 h before imaging. The nanoparticles were synthesized as reported by Dossi et al. (2013). While GFP provided a green fluorescence signal for viable cells, the rhodamine‐labeled nanoparticles allowed the detection of the total cells (viable + dead).

#### 2.1.2 2D Monolayer Cultures and 3D Bioprinted Constructs

To assess model performance under different spatial conditions and signal distributions, images were acquired from 2D and 3D sample configurations. For 2D monolayer cultures, a sterile ink was prepared by dissolving 5% w/v GelMA and 0.25% w/v photoinitiator (LAP) in sterile cell culture medium at 37°C under stirring. This ink was then crosslinked under UV light (365 nm, 60 s) to form a thin hydrogel layer at the bottom of each well of a 48‐well plate. HUVECs were then seeded at a density of 30,000 cells/cm^2^ on top of the GelMA layer and routinely grown in endothelial cell growth medium as reported in Section 2.1.1.

For 3D experiments, cells were recovered after expansion by trypsinization (Trypsin–EDTA solution, Sigma‐Aldrich, St. Louis, USA), pelleted by centrifugation (Centrifuge 5810 R, Eppendorf, Germany), and resuspended in culture medium. After counting, the suspension was adjusted to the desired cell concentration, i.e., 50 *×* 10^6^ cells/mL. A GelMA ink, with the same composition as reported for 2D cultures, was pre‐warmed to 37°C to ensure proper handling and mixed with the cell suspension at a ratio of 9:1 GelMA:cell suspension, yielding a final concentration of 5 *×* 10^6^ cells/mL in the bioink. The bioink was loaded into a cartridge and printed into grid‐like structures with a final thickness of approximately 800 μm using an extrusion‐based bioprinter (CELLINK BIOX, Sweden). After printing, the different constructs were crosslinked by exposure to UV light (365 nm, 60 s) at room temperature and subsequently incubated under standard culture conditions.

In addition to bioprinted constructs for model training, a set of samples was specifically prepared for validation. For this, HUVECs were detached, counted, and bioprinted in GelMA constructs at a density of 30,000, 50,000, and 100,000 cells/cm^2^. The 3D constructs were cultured under standard conditions and imaged at different time points. These images were used for qualitative comparison between manual annotations and automated predictions, as described in Section 3.6.

Both 2D and 3D samples were stained using the same protocol and imaged using identical microscope settings after 1, 3, and 7 days to monitor viability over time. This setup allowed for the evaluation of model performance under different spatial arrangements and fluorescence intensities.

#### 2.1.3 Image Acquisition

Images were acquired using the Celena S digital microscope (Logos Biosystems, South Korea). For each sample, images were acquired in triplicate in three channels: brightfield, green, and red fluorescence. For green fluorescence signal, a LED filter cube with excitation wavelength 470 nm and bandwidth 30 nm and emission wavelength 530 nm and bandwidth 50 nm was used. For the red fluorescence signal, the installed filter allowed excitation at 530 nm with bandwidth 40 nm and emission at 605 nm with bandwidth 55 nm. Acquisitions were performed at magnifications 10*×* and 20*×* with a resolution of 1280 *×* 1024 pixels. Brightness and contrast settings were manually adjusted before imaging. The exposure time was automatically adjusted for each acquisition from the imaging software of the microscope. Once established, the same microscope settings were maintained for all acquisitions to ensure consistency among images. The same acquisition settings were maintained during image collection, and the fluorescence intensity thresholds used for cell classification were applied uniformly to all images without image‐specific adjustments. All three channels were used for segmentation to exploit the information provided by both fluorescence and structural details in the brightfield channel.

#### 2.1.4 Ground Truth Labeling

A total of 42 2D images were manually annotated for training and testing. These where then randomly subdivided in two datasets. Specifically, 36 images were used for training, while 6 were reserved for testing. The images were manually labeled by the author using ImageJ (FIJI) software, assisted by the magic wand tool to select individual cells, followed by manual refinement where necessary. Binary masks separating cells from the background were produced as ground truth references for training and testing.

### 2.2 Preprocessing

Before training, images were enhanced using the *rolling ball* algorithm to improve contrast between background and cells, facilitating segmentation. Images were then divided into patches of size 512 *×* 512 pixels with a stride of 256 pixels. Several data augmentation techniques were applied to the images and their corresponding masks using the Albumentations library to improve the model robustness and generalization ability. These included random horizontal and vertical flipping, random rotations, and the *ShiftScaleRotate* transformation, which performs random shifts, zooming, and rotations. After this process, a total of 1296 images were obtained for training, and 216 for testing. Then, channel‐wise normalization was performed to standardize input data and facilitate model convergence during training. For each of the three input channels (brightfield, green fluorescence, and red fluorescence), grayscale images were converted to tensors, and the mean (*μ*) and standard deviation (*σ*) of pixel intensity values were computed across the training dataset. These values were then used to normalize each image according to Equation (1).

(1) $$\text{Normalized Pixel}=\frac{\text{Pixel Intensity}-\mu }{\sigma }$$

### 2.3 Hyperparameter and Patch Size Selection

A patch size of 512 *×* 512 pixels with a stride of 256 pixels was adopted after testing multiple combinations (512 *×* 512, 320 *×* 320 pixels; stride of 512, 320, 256 pixels). The hyperparameters were selected based on a tradeoff between computational time and quality of the segmentation, quantified through the binary cross‐entropy (BCE) in validation as loss function. A training limit of 100 epochs was chosen to ensure sufficient time for convergence, while early stopping (patience = 5) was implemented to avoid overfitting and reduce training time. The initial learning rate was set to 1 *×* 10^−5^. Batch sizes of 4, 8, 16, and 32 were tested.

### 2.4 Segmentation

For the segmentation task, the *U* ^2^‐Net architecture was used. The model was initialized with random weights and trained from scratch using the three input channels along with their corresponding ground truth binary masks. The dataset was divided into two subsets: 80% of the images were used for training, and 20% were reserved for validation. The validation set was used to monitor performance during training and ensure the model generalized well to unseen data. The model was trained using the previously defined hyperparameters and the Adam optimizer, which is particularly suited for problems with sparse gradients and noisy data. BCE was used as the loss function, commonly applied for binary segmentation tasks. Training and validation losses were tracked at each epoch to monitor convergence and detect overfitting. Additionally, the Dice score was computed at the end of each epoch for both training and validation datasets as the overlap between ground truth labels and model results as in Equation (2) (Seghier 2024).

(2) $$\text{Dice score}=\frac{2\mathrm{TP}}{2\mathrm{TP}+\mathrm{FP}+\mathrm{FN}}$$

where TP are the true positive results, which is the number of counts correctly predicted by the model; FP are the false positives, i.e., cells wrongly identified by the model; FN are the false negatives, i.e., cells labeled manually and not recognized by the model. To ensure optimal performance, the final model used for evaluation was selected based on the epoch with the highest validation Dice score. After training, the model was tested on a separate test set comprising 216 image patches derived from six full‐resolution 2D images not used during training or validation. The Dice coefficient was computed for each batch in the test set.

### 2.5 Post‐Processing and Viability Assessment

After segmentation, the resulting binary masks underwent a post‐processing step to identify live and dead cells. First, masks were refined by excluding regions with fluorescence intensities (in both red and green channels) below a predefined threshold, with the aim of removing background noise and artifacts not related to cells. Next, a watershed algorithm was applied using the *watershed* function of the skimage library to separate touching or overlapping cells. Following segmentation, each labeled region in the binary mask was analyzed. A minimum area threshold of 50 pixels^2^ was set, as this value is below the expected area covered by a single cell at 10× magnification, thus allowing the removal of small artifacts. For the remaining regions, mean fluorescence intensities in the red and green channels were computed. Classification was then performed based on these intensity values: regions with both red and green signals were classified as live cells; regions with only red signal were classified as dead cells. Specifically, a segmented region was classified as a live cell when the mean green fluorescence intensity exceeded 8 (8‐bit grayscale). On the other side, no threshold was set on the red channel, because the rhodamine fluorescence signal was consistently weaker than the green fluorescence one. The selected thresholds were applied consistently to all images without image‐specific adjustment.

### 2.6 Validation and Evaluation

To validate the model ability to generalize to unseen data and evaluate its performance in practical applications, a set of 70 images from 2D monolayer cultures was used. Each image was manually counted three times, and the average of these counts was used as a reference value for comparison with the model counts. This approach aimed to minimize human error and provide a more consistent ground truth for evaluating model accuracy. Performance was assessed by calculating different statistical metrics commonly used for regression tasks. In particular, the Mean Absolute Error (MAE), Root Mean Square Error (RMSE), Coefficient of Determination (*R* ^2^), and the Pearson correlation coefficient (*r*) were adopted for live and total cells. For 3D experiments, quantitative validation against manual annotations was not feasible due to the intrinsic complexity of manually segmenting and counting cells within volumetric constructs. Instead, the evaluation focused on qualitative and application‐oriented validation. Model outputs were visually inspected across multiple regions of interest to qualitatively assess segmentation accuracy and robustness. Furthermore, model counts in GelMA‐based constructs were compared with manual annotations in a case study of 3D scaffolds (see Section 3.6).

### 2.7 Implementation Details

The pipeline was implemented in Python 3.12.4. The core libraries used in this work included PyTorch (version 2.6.0) for model definition and training, Torchvision (version 0.21.0) for dataset handling and transformations, and Albumentations (version 2.0.4) for data augmentation. Image processing relied on OpenCV (version 4.11.0.86), Scikit‐image (version 0.25.2), SciPy (version 1.15.1), and PIL (version 11.1.0). Data analysis and visualization were performed using NumPy (version 2.2.2), Pandas (version 2.2.3), Seaborn (version 0.13.2), and Matplotlib (version 3.10.0). The *U* ^2^‐Net architecture was imported from a local implementation of the original model repository (source: https://github.com/xuebinqin/u‐2‐net). All training and inference operations were run on a CPU system with 64.0 GB of RAM. No GPU was used.

## 3 Results and Discussion

### 3.1 Dataset Generation

In this work, green fluorescent HUVECs were selected for model development and testing. These were incubated with rhodamine B‐labeled polymethyl methacrylate nanoparticles for a red staining before seeding. This strategy provided dual fluorescence microscopy trackability: the green channel could in fact be exploited to detect viable cells, while the red channel was considered representative of both live and dead cells. Images of the cell cultures were acquired at 10*×* and 20*×* magnifications for both 2D monolayer cultures and 3D bioprinted constructs across three channels: brightfield, green fluorescence (GFP), and red fluorescence (rhodamine‐labeled nanoparticles). For 3D constructs, images were captured at different focal planes along the z‐axis to account for the sample thickness. Out‐of‐focus signals from cells outside the selected focal plane were excluded during post‐processing through threshold‐based filtering, ensuring that only well‐focused cells were considered for subsequent analysis. As shown in Figure 2 images typically present isolated or slightly clustered cells with well‐defined outlines, while 3D constructs show complex spatial distributions and overlapping signals due to cellular stacking and gel depth.

**FIGURE 2:**
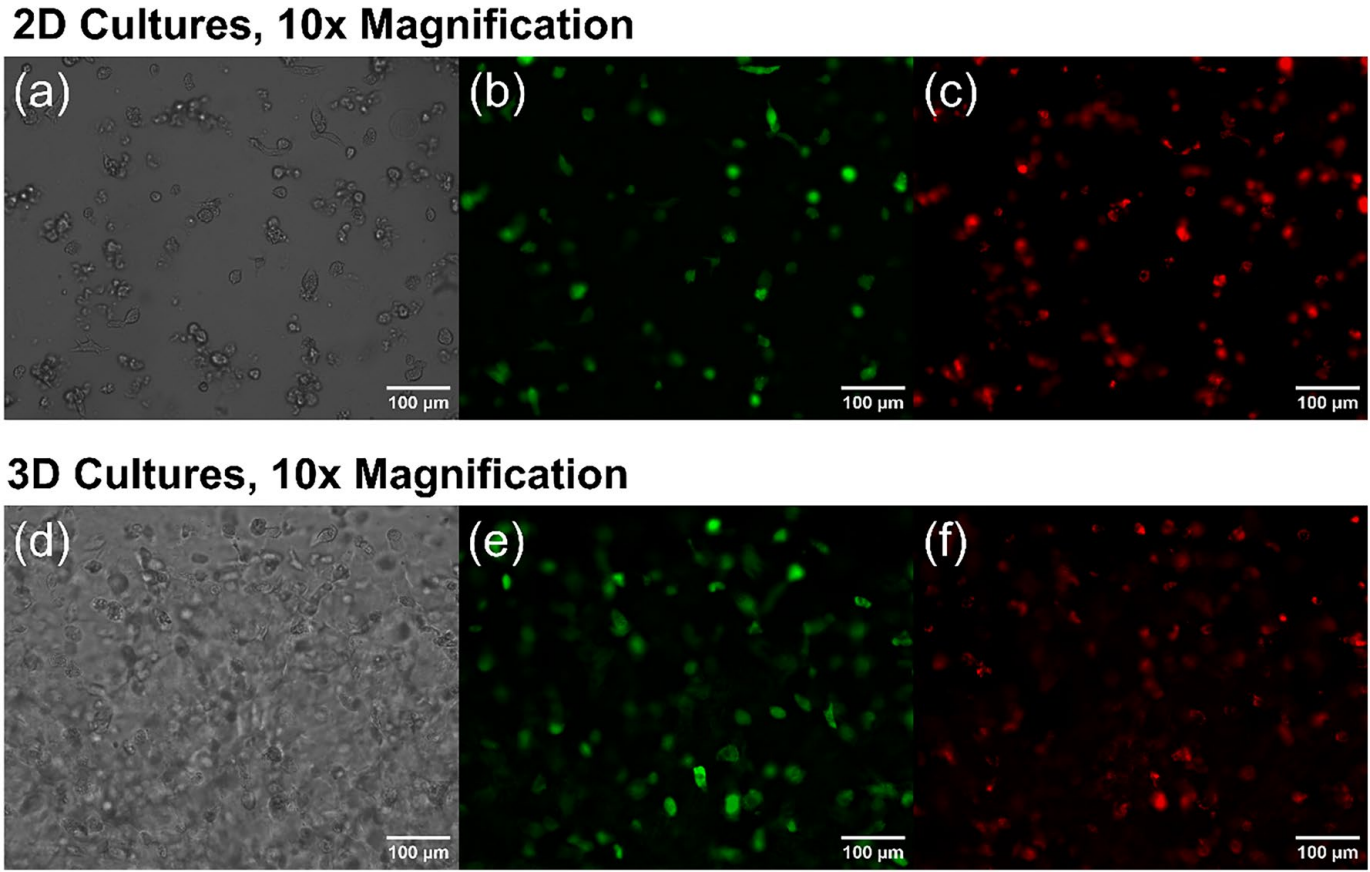
Example of 2D (a−c) and 3D (d−f) images at 10*×* magnification for the three microscope channels: Brightfield (a, d), green (b, e), and red fluorescence (c, f). Scale bar: 100 μm.

Ground truth binary masks were generated through manual annotation of the brightfield channel. Figure 3 shows sample input images alongside their corresponding manually labeled masks.

**FIGURE 3:**
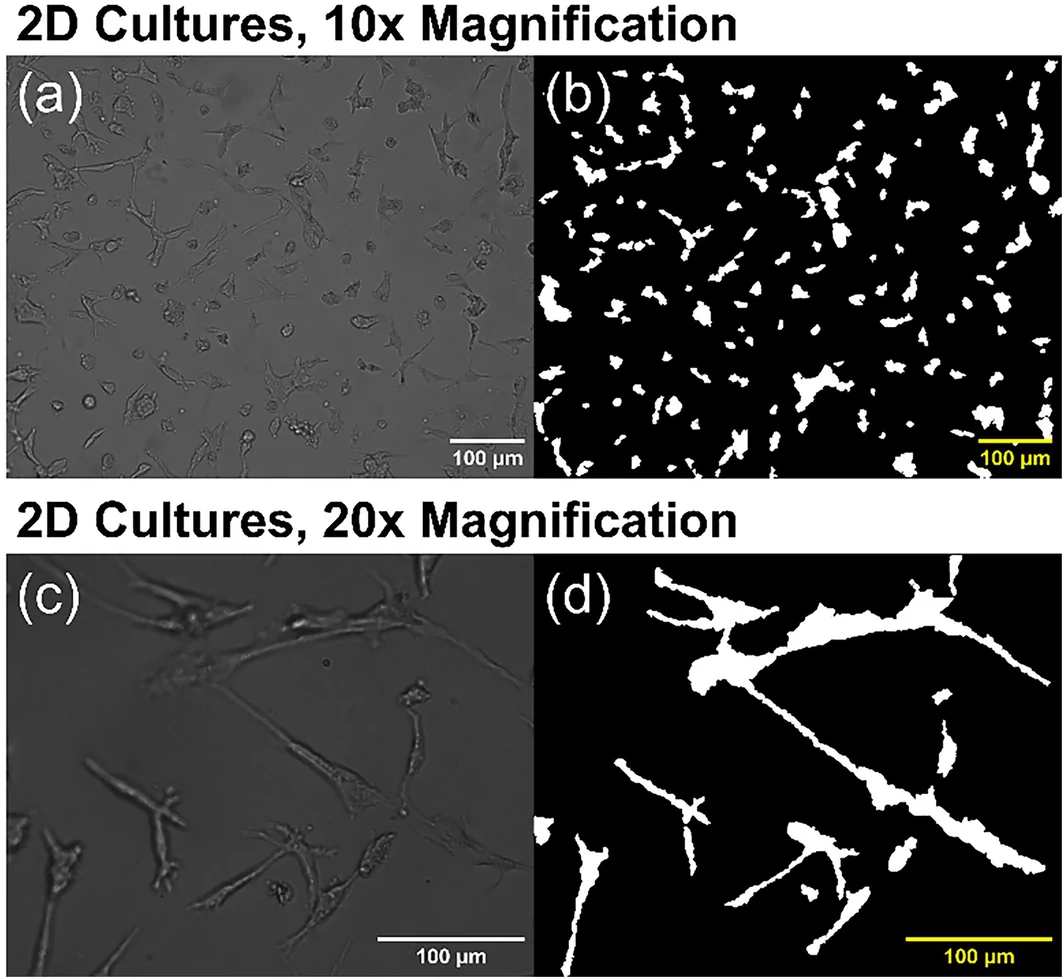
Example of the original brightfield images (a, c) and manually annotated ground truth labels (b, d) at 10*×* (a, b), and 20*×* (c, d) magnifications. Scale bar: 100 μm.

### 3.2 Hyperparameters and Patch Size Selection

To identify the most effective patching strategy, different combinations of patch size and stride were tested. As shown in Table 1, the configuration using 512 *×* 512 pixel patches with a 256‐pixel stride yielded the highest Dice score (0.7403), outperforming both smaller patches and configurations with larger strides.

**TABLE 1:** Performance of different patch size and stride combinations based on Dice score. Dice scores were obtained during early experiments before final preprocessing steps (e.g., normalization) were added. Final model performance is reported in Section 3.4.

Patch size	Stride	Dice score
[pixels]	[pixels]	[−]
512 *×* 512	512	0.7057
512 *×* 512	256	0.7403
320 *×* 320	320	0.6901

The hyperparameter values were selected through iterative testing, aiming to balance segmentation accuracy, training efficiency, and hardware limitations. All experiments were performed on a CPU‐based workstation without GPU acceleration. Table 2 reports the results of this analysis performed at fixed learning rate of 1 *×* 10^*−*5^ and at different batch size.

**TABLE 2:** Segmentation performance in terms of loss function on the validation dataset and computational time required for model training for different batch sizes. In all the cases, a learning rate of 1 *×* 10^−5^ and a maximum number of epochs of 100 were set. For the latter, an early‐stop criterium with patience = 5 was introduced.

Batch size	Validation loss	Computational time	Epochs
[−]	[−]	[h]	[−]
4	0.7198	18	30
8	0.7099	28	43
16	0.6819	36	33
32	0.6836	59	45

The loss initially decreased with the batch size, indicating a progressive increase in the segmentation performance. However, this is accompanied by a decisive increase in the time required for model training. Among the tested values, a batch size of 16 provided the best compromise between computational time and performance and was therefore selected for the following tasks.

### 3.3 Segmentation Results

By selecting these parameters, the training and validation loss and Dice score over 70 epochs are shown in Figure 4a,b. Although the training limit was set to 100 epochs, early stopping (patience = 5) was applied to prevent overfitting and reduce unnecessary computation, which resulted in training stopping at epoch 70. The loss curves indicate consistent convergence, with both training and validation loss decreasing steadily. However, the validation loss reaches a plateau earlier, suggesting potential overfitting after approximately 30 epochs. The segmentation performance improves rapidly during the initial epochs, as confirmed by the steep decrease in the loss function and increase in the Dice score. However, while the Dice score for the training steadily increases, the same metric referred to the validation dataset reaches a plateau and shows minor fluctuations after epoch 20. The observed gap between training and validation Dice scores reflects a modest overfitting trend. To address this, the final model used for evaluation was selected based on the highest validation Dice score, corresponding to epoch 65, where it reached a value of 0.8433, indicating effective segmentation performance.

**FIGURE 4:**
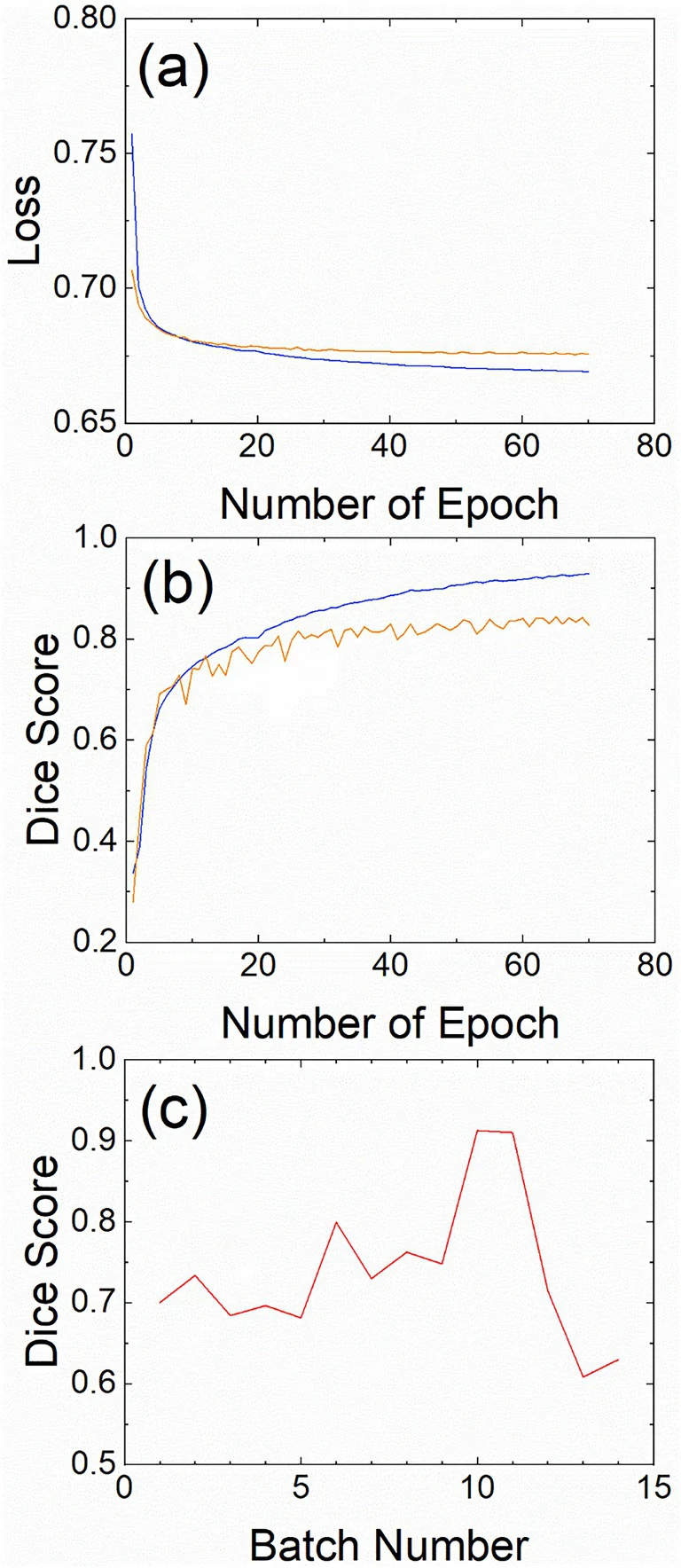
(a) Loss and (b) Dice score convergence curves for both training (blue) and validation (orange) datasets. (c) Dice score computed on an additional test set, showing model performance on unseen image patches.

The model was further evaluated on an independent test set, whose Dice score is shown in Figure 4c. The average Dice score was 0.7403, slightly lower than the validation performance. This suggests a certain degree of batch‐to‐batch variability in terms of model performance, even though the difference is quite limited.

It is worth mentioning that training and validation were performed on a total of 42 manually annotated images, augmented by random rotations and flipping. On one hand, this allowed to improve the model robustness. On the other side, the diversity of the images remains limited to the original ones and needs to be stressed. At the same time, the splitting between training and validation images was performed before data augmentation, which ensured no cross contamination of the two datasets.

The trained *U* ^2^‐Net produced binary segmentation masks from the three‐channel input images. Figure 5 shows representative examples of input images and their corresponding predicted segmentation masks. Visual inspection confirmed that the segmentation was generally reliable, correctly capturing cell boundaries across both 2D and 3D samples. The model was particularly accurate in 2D monolayers, where cells appeared isolated or moderately clustered with sharp outlines. In contrast, segmentation in 3D constructs was more challenging due to overlapping signals, variations in fluorescence intensity, and the presence of partially out‐of‐focus regions. In these cases, the model occasionally misinterpreted the boundaries between touching cells or struggleds to distinguish low‐contrast regions clearly. Nevertheless, the integration of information from the three fluorescence channels allowed the network to retain good robustness, even in more complex 3D contexts.

**FIGURE 5:**
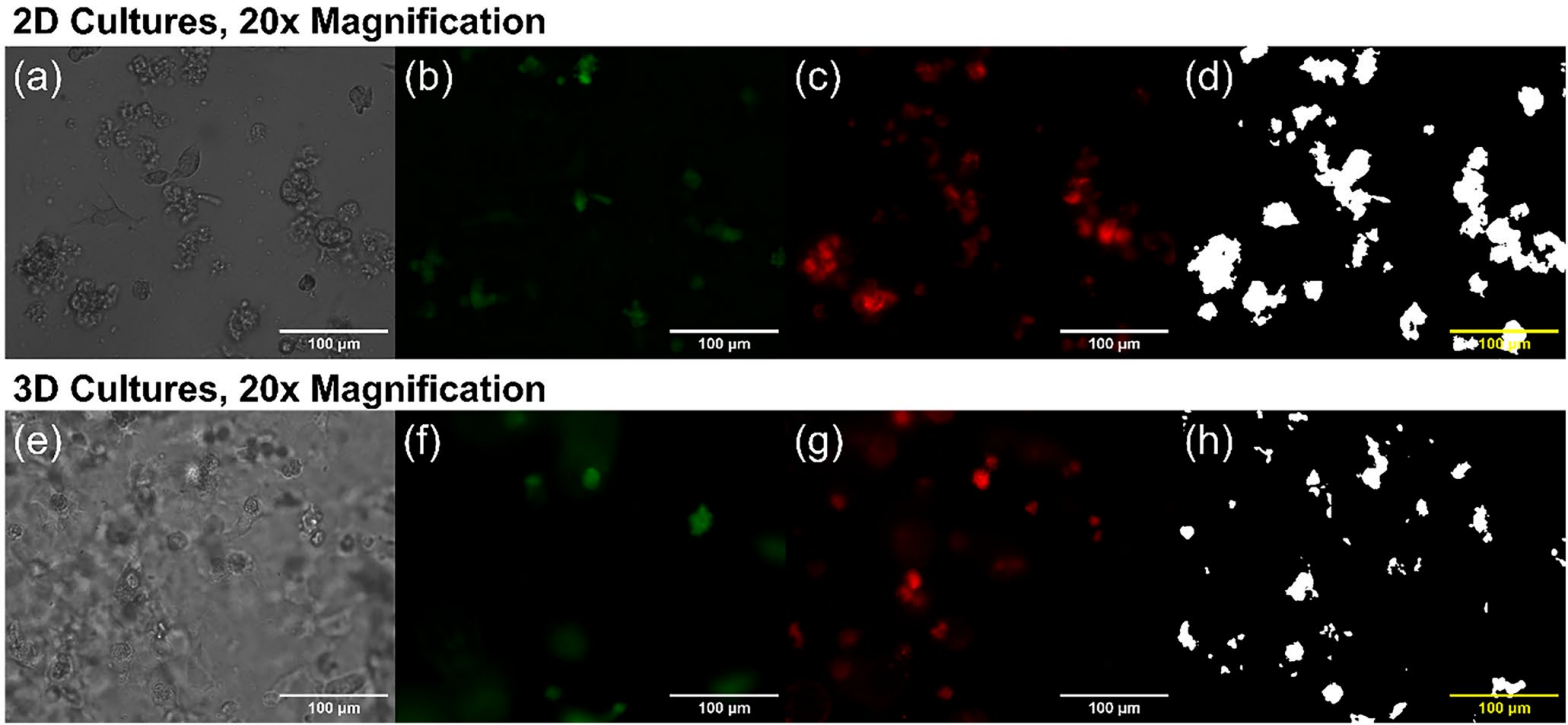
Examples of three‐channel inputs: Brightfield (a, e), green fluorescence (b, f), red fluorescence (c, g), and corresponding predicted segmentation masks (d, h) for both 2D (a−d) and 3D (e−h) datasets at 20*×* magnification. Scale bar: 100 μm.

### 3.4 Post‐Processing and Classification

Following segmentation, post‐processing steps were applied to separate individual cells and classify them as live or dead based on fluorescence intensity thresholds (see Section 2.5). Figure 6 illustrates the refined binary mask, watershed‐separated objects, and the final classification map distinguishing live (green) and dead (red) cells for the same images reported in Figure 5.

**FIGURE 6:**
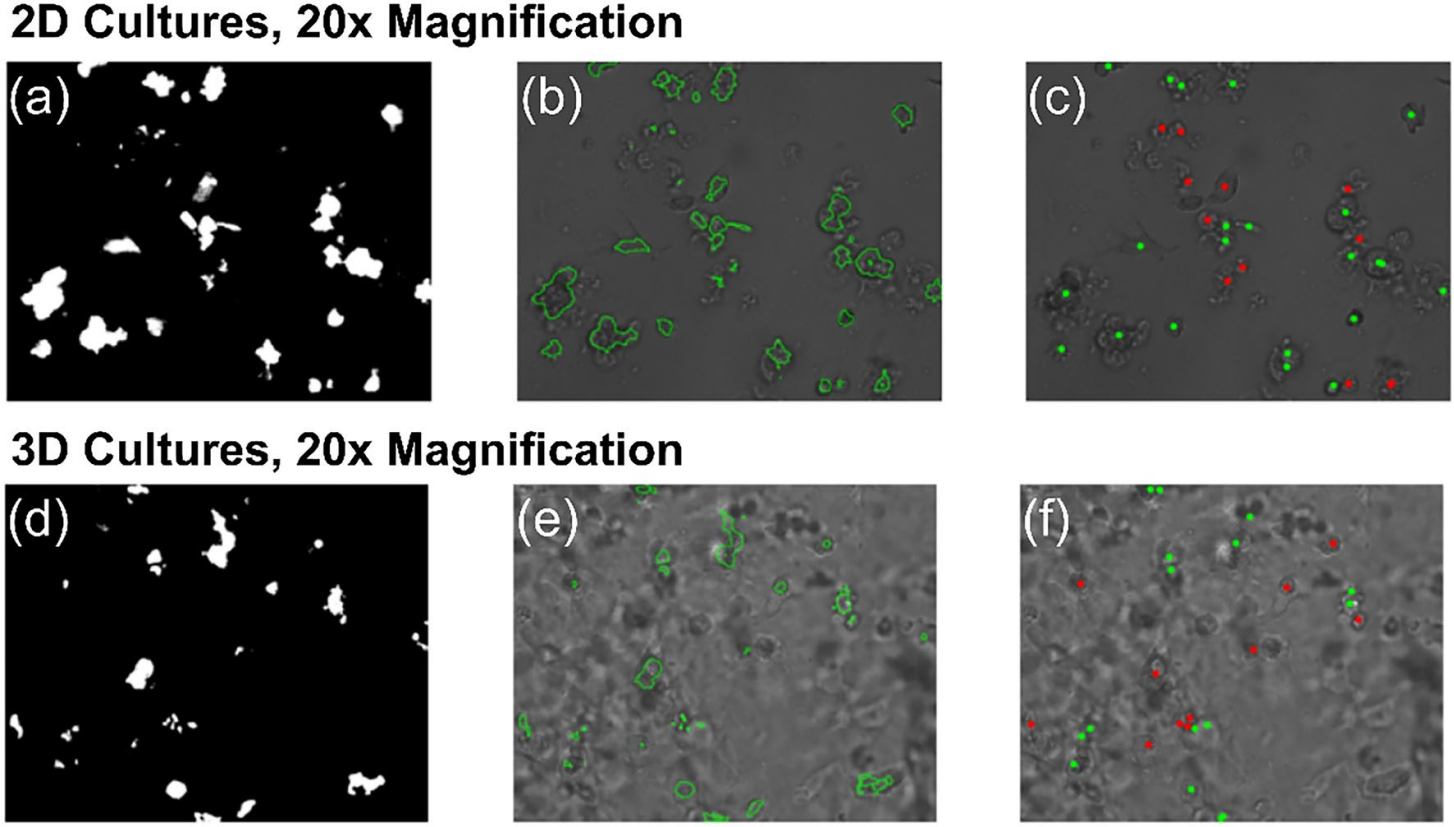
Post‐processing stages for representative binary images (a, d), refined watershed output (b, e), and final classification in live (green) vs. dead cells (red) (c, f) for 2D (a−c) and 3D datasets (d−f).

As visible comparing Figures 5 and 6, the binary mask was refined after post‐processing by removing regions with green and red fluorescence intensities below a defined threshold (see Section 2.5), which were considered as background noise. The intensity thresholds were chosen through iterative testing, selecting values that effectively excluded out‐of‐focus signals and weak background fluorescence while preserving cellular regions.

Moreover, regions below a minimum area threshold of 50 pixels^2^ were excluded from the count. This threshold was set smaller than the average size of a single cell at 10x magnification to remove imaging artifacts without discarding real cells.

As can be observed, some regions of overlapped cells are not perfectly recognized and divided by the watershed algorithm. Although there is room for improvement in separating overlapping cells, segmentation quality is limited by image resolution and signal quality. However, from a visual inspection, the majority of the cells appear to be correctly labeled.

### 3.5 Model Validation and Evaluation

After having developed the algorithm able to differentiate and automatically count live and dead cells, the model performance was quantitatively validated on a manually annotated set of 70 2D images. Manual counts were performed three times per image, and the mean count was used as the reference. This approach aimed to reduce variability due to subjectivity and human error and ensure more reliable annotations. While 3D samples were also segmented and analyzed by the model, manual annotation of 3D data was not considered for model validation due to the complexity and subjectivity of manual cell counting in 3D images. On the other side, the model results on 3D samples were compared to manual counts for a specific case study of bioprinted cells in the following Section.

The model reliability was evaluated considering four metrics: MAE, RMSE, determination coefficient (*R* ^2^), and Pearson correlation coefficient (*r*), focusing on live and total cell counts, which are the direct outputs from the model, assessed by evaluating green and red labeled cells, respectively. Metrics for dead cells and vitality were not evaluated separately, as these values are directly derived from live and total cell predictions (i.e., Dead = Total − Live; Vitality = Live/Total). Therefore, their accuracy is implicitly included in the reported results. The results of this analysis are summarized in Table 3.

**TABLE 3:** Quantitative error metrics on live and total cell counts predicted by the model and on those evaluated from ImageJ with respect to manual counts for 2D dataset.

	Live cells from model	Total cells from model	Live cells from ImageJ	Total cells from ImageJ
MAE [cells/image]	2.42	3.63	4.39	12.30
RMSE [cells/image]	3.70	5.44	6.33	19.76
*R* ^2^ [−]	0.98	0.96	0.92	0.49
*R* [−]	0.99	0.98	0.97	0.96

These results indicate strong agreement between the model predictions and manual annotations. High *R* ^2^ values (0.98 and 0.96) and Pearson correlation coefficients (0.99 and 0.98) reflect excellent predictive performance and linear correlation. The low MAE and RMSE values show that numerical errors are also minimal, especially in live cell predictions, which are of particular interest in most biological analyses. Figure 7a,b shows parity plots comparing predicted versus manual counts, which further confirm the good predictivity of the model on both live and total cells, with no significant outliers.

**FIGURE 7:**
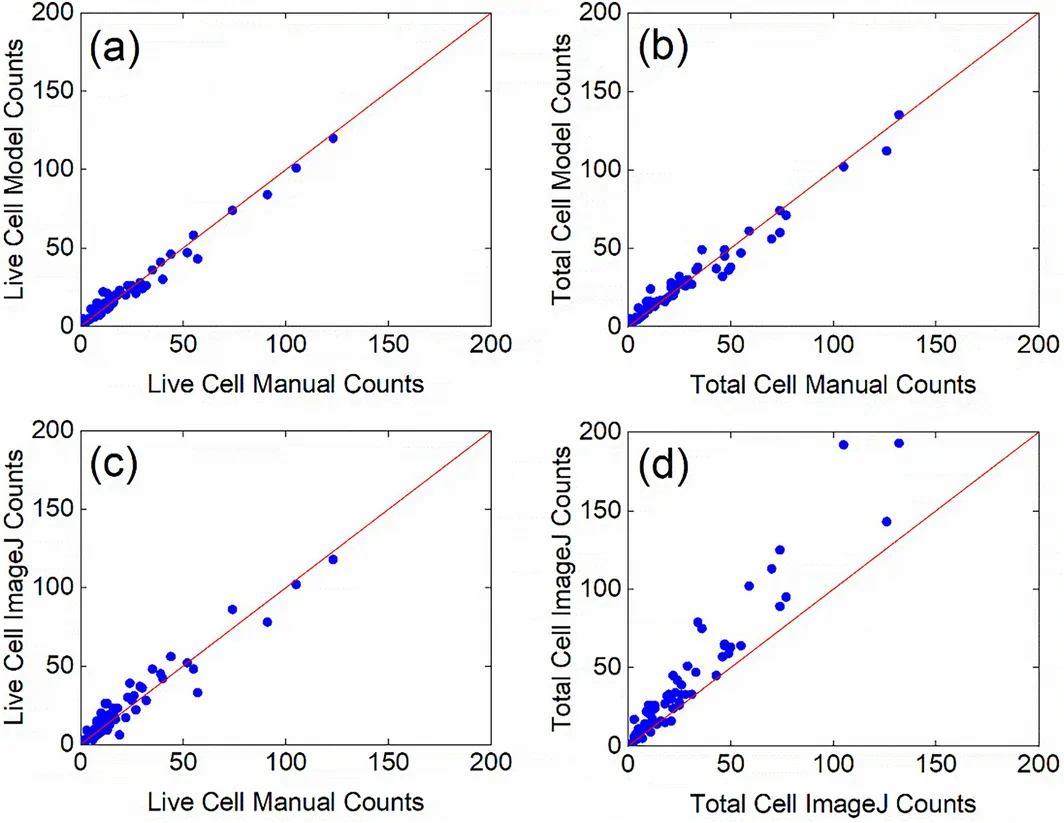
Parity plots comparing predicted vs. manual counts for live (a) and total (b) cells and ImageJ vs. manual counts for live (c) and total (d) cells.

In addition to manual validation, the model performance was benchmarked against ImageJ, a widely used tool in fluorescence microscopy analysis. The same set of 70 2D images was processed in ImageJ using standard thresholding and watershed segmentation techniques, and the resulting live and total cell counts were compared to manual annotations. Table 3 and Figure 7c,d report the resulting error metrics and parity plots from this comparison. The deep learning model developed in this work shows improved performance in both live and total cell counts, achieving higher *R* ^2^ and Pearson correlation values (0.98 vs. 0.92 for live, and 0.96 vs. 0.49 for total) and lower error values compared to ImageJ. The important gain in accuracy is evident in particular when comparing the total cell counts (Figure 7b,d). While the model provides results in great agreement with the manual counts, the ImageJ outputs strongly deviate, in particular when evaluating a large number of cells. These results underline the advantages of the model not only in terms of accuracy, but also efficiency and reproducibility. The deep learning pipeline allows for fully automated analysis without manual tuning or human intervention, which is an essential feature for scaling up biological experiments and reducing analysis time and subjectivity.

### 3.6 Model Application to 3D Constructs

To assess the performance of the proposed model in a 3D application, cell‐laden GelMA‐based constructs were printed with three different cell densities: 30,000, 50,000, and 100,000 cells/cm^2^. The number of total cells and live cells was then tracked over time by acquiring and analyzing images at three time points: Day 1, 3, and 7. For comparison, live and total cells were also manually counted.

Figure 8 presents an example of image segmentation alongside a histogram showing the total number of cells (expressed in cells/mm^2^) and viability (determined as ratio live cells/total cells) over time, for both manual counting and model counting. Even if some variability is present in the absolute cell numbers, likely due to the inherent subjectivity and limitations of manual annotation, the values obtained from the model closely follow the same trend as the manual counts across all time points and conditions. Importantly, the segmentation remains accurate even for cells that are barely distinguishable by eye, demonstrating the sensitivity, and reliability of the model.

**FIGURE 8:**
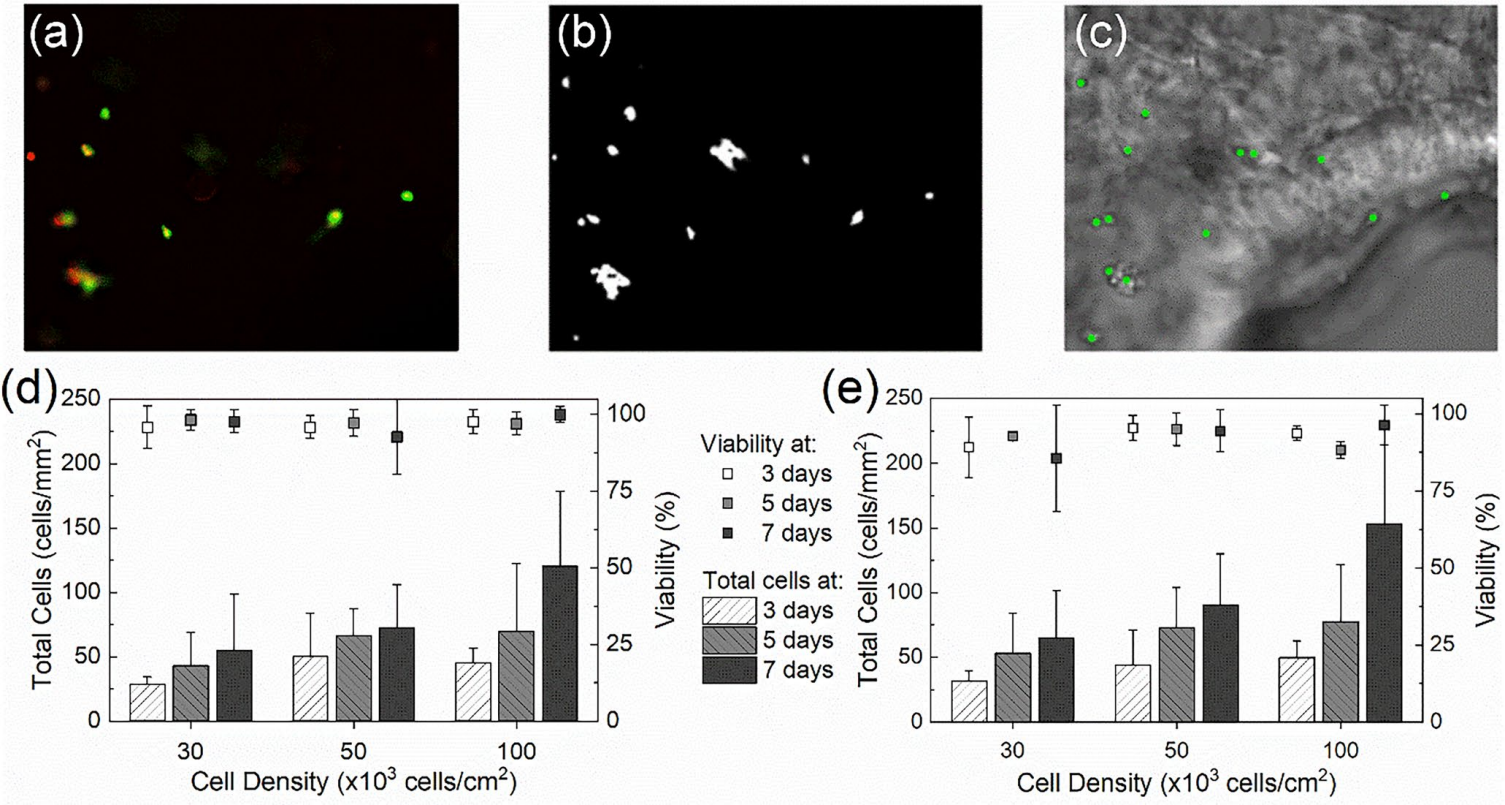
Example images of cells in 3D constructs at 10× magnification, 30,000 cells/cm^2^ seeding density. (a) Green and red fluorescence overlay, (b) model‐predicted segmentation, and (c) live/dead cell counts derived from the model. (d) Histograms illustrating the distribution of total cells (bars) and viability (squares) across days for different cell densities (30,000, 50,000, 100,000 cells/cm^2^) as evaluated manually, and (e) automatically counted in 3D samples. Cell densities were calculated by normalizing the counted cells to the analyzed image area and are reported as mean ± standard deviation from three replicates.

To quantitatively evaluate the agreement between the two counting methodologies, a paired analysis was conducted across all 27 experimental observations (three replicates for each condition). The automated model showed a strong correlation with manual counts, yielding Pearson correlation coefficients of r=0.9631 (p<0.001) for live cells and r=0.9665 (p<0.001) for total cells, with corresponding $R^{2}$ of 0.8714 and 0.8684, respectively. The accuracy of the automated counts was further supported by low MAE and RMSE values, equal to 4.85 and 6.96 cells/image for live counts and 5.22 and 7.10 cells/image for total counts, respectively. A minor systematic underestimation was observed (mean bias of −3.22 cells/image for live cells and −4.19 cells/image for total cells), which may be partly attributable to overlapping cells in complex 3D regions.

This example demonstrates the practical utility of the developed model for real‐world applications. By automating the cell viability assessment, the model significantly reduces the time and effort required for manual quantification, offering a valid alternative for routine biological experiments.

## 4 Conclusions

With the increasing attention to 3D bioprinting in biomedical research, the need for reliable methods to assess cell viability in complex 3D constructs has become increasingly important. Automated analysis in such structures is challenging due to signal attenuation, optical transparency, and out‐of‐focus interference. This study presents a hybrid, fully automated pipeline that integrates deep learning and traditional image processing to enable accurate segmentation and viability assessment in fluorescence microscopy images. The proposed approach offers a valid alternative to manual or semi‐automated workflows, reducing manual workload. For segmentation, the *U* ^2^‐Net architecture was trained from scratch, with hyperparameters such as batch size, learning rate, and patch size optimized through iterative testing to find a balance between segmentation accuracy and computational efficiency. The final configuration, using a patch size of 512 *×* 512 pixels with a stride of 256 pixels, demonstrated strong performance, with a validation Dice score of 0.8433. The training process showed consistent convergence and minimal overfitting, as indicated by both Dice score and BCE loss. Model performance was quantitatively evaluated by comparing both manual and ImageJ‐based counts through the evaluation of multiple metrics (MAE, RMSE, *R* ^2^, and Pearson's correlation coefficient). The results of this analysis indicated improved accuracy in predicting the number of cells, particularly for total cell counts. To showcase the model applicability in a real experiment, the cell density was tracked during time in GelMA‐based 3D constructs. Automated cell counts were compared with manual annotations. While minor discrepancies in absolute cell numbers were observed, likely due to variability in manual counting, the model accurately captured temporal trends in cell proliferation. In summary, the proposed pipeline is a reliable and efficient tool for cell segmentation and viability analysis from fluorescence microscopy images. It offers a solution for high‐throughput workflows, particularly where manual annotation is infeasible or error‐prone.

## Author Contributions

**Francesco Iannacci:** investigation, methodology. **Bianca Maria Colosimo:** funding acquisition, writing – review and editing. **Federica Valtellina:** data curation, writing – original draft, investigation, software. **Mattia Sponchioni:** funding acquisition, supervision, writing – review and editing, methodology.

## Funding

This work was supported by Fondazione Cariplo (2021‐1518).

## Conflicts of Interest

The authors declare no conflicts of interest.

## Data Availability

The data that support the findings of this study are available from the corresponding author upon reasonable request.
